# The roles of Mesp family proteins: functional diversity and redundancy in differentiation of pluripotent stem cells and mammalian mesodermal development

**DOI:** 10.1007/s13238-015-0176-y

**Published:** 2015-06-19

**Authors:** Qianqian Liang, Chen Xu, Xinyun Chen, Xiuya Li, Chao Lu, Ping Zhou, Lianhua Yin, Ruizhe Qian, Sifeng Chen, Zhendong Ling, Ning Sun

**Affiliations:** Department of Physiology and Pathophysiology, School of Basic Medical Sciences, Research Center on Aging and Medicine, Shanghai Medical College, Fudan University, Shanghai, 200032 China; Department of Surgery, The Branch of Shanghai No. 1 Hospital, School of Medicine, Shanghai Jiaotong University, Shanghai, 200081 China

**Keywords:** Mesp, transcription factor, pluripotent stem cells, cardiovascular differentiation, somitogenesis

## Abstract

Mesp family proteins comprise two members named mesodermal posterior 1 (Mesp1) and mesodermal posterior 2 (Mesp2). Both Mesp1 and Mesp2 are transcription factors and they share an almost identical basic helix-loop-helix motif. They have been shown to play critical regulating roles in mammalian heart and somite development. Mesp1 sits in the core of the complicated regulatory network for generation of cardiovascular progenitors while Mesp2 is central for somitogenesis. Here we summarize the similarities and differences in their molecular functions during mammalian early mesodermal development and discuss possible future research directions for further study of the functions of Mesp1 and Mesp2. A comprehensive knowledge of molecular functions of Mesp family proteins will eventually help us better understand mammalian heart development and somitogenesis as well as improve the production of specific cell types from pluripotent stem cells for future regenerative therapies.

## INTRODUCTION

**Figure 1:**
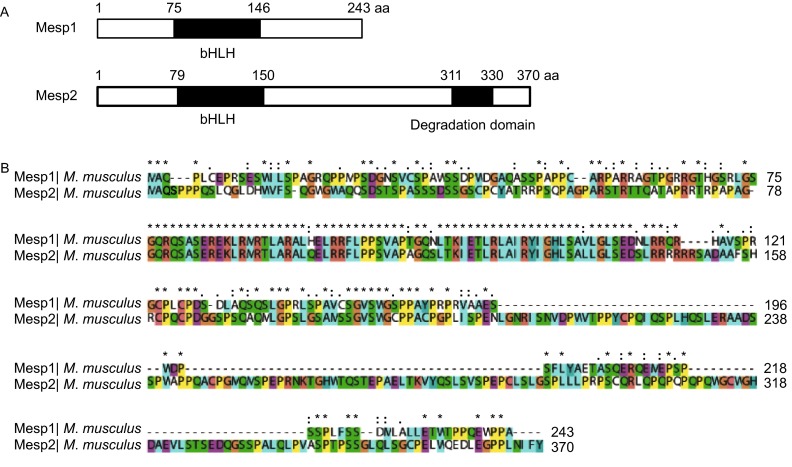
**Similarities between mouse Mesp1 and Mesp2**. (A) Schematic diagram of mouse Mesp1 and Mesp2. The bHLH domain is colored black. (B) Alignment of mouse Mesp1 and Mesp2 shows that the bHLH domains are almost identical. The alignment was done using Clustal X. Conserved positions are marked by characters above the alignment: “*” indicates identical residue. “:” shows that one of the following ‘strongly conserved’ groups: STA, NEQK, NHQK, NDEQ, QHRK, MILV, MILF, HY or FYW. “.” shows that one of the following ‘weaker conserved’ groups: CSA, ATV, SAG, STNK, STPA, SGND, SNDEQK, NDEQHK, NEQHRK, FVLIM or FYM

In humans and mice, Mesp family proteins comprise two members named mesodermal posterior 1 (Mesp1) and mesodermal posterior 2 (Mesp2), which have been shown to play critical roles in embryonic mesodermal development. Both Mesp proteins are transcription factors and share an almost identical basic helix-loop-helix (bHLH) motif (Fig. 1). Mesp1 and Mesp2 genes are located on the same chromosome, head to head, and are separated only by ~25 kb in human and ~16 kb in mouse, respectively. Further, both Mesp proteins are expressed in the early mesoderm in embryos, suggesting similar functions in embryonic development. However, single gene knockout studies in mice indicated the two Mesp proteins exhibit considerable functional diversity. Here we discuss the similarities and differences in the molecular functions of Mesp1 and Mesp2 in mammalian mesodermal development and possible future research.

## FUNCTIONAL ROLE OF MESP1 IN EMBRYONIC MESODERMAL DEVELOPMENT

**Figure 2:**
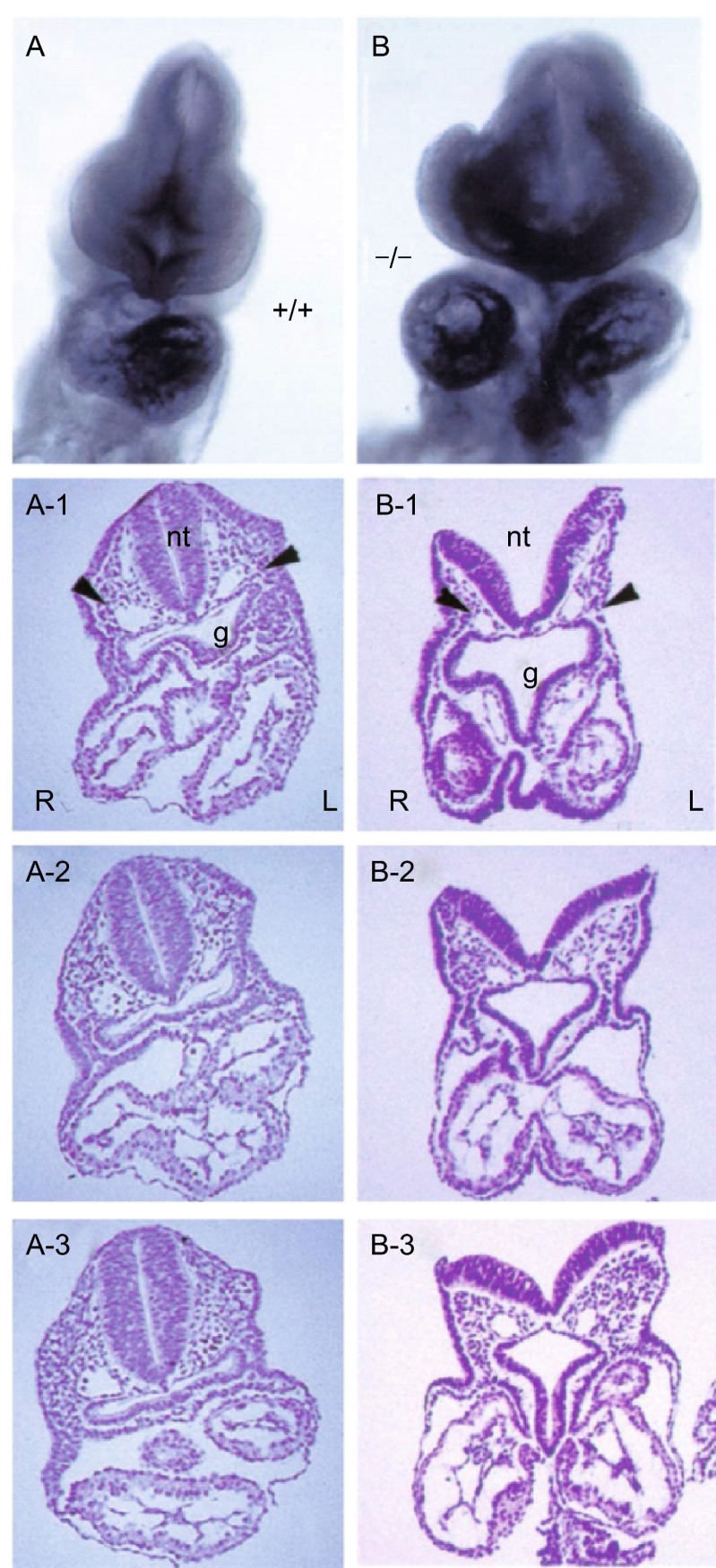
**Mesp1-null embryos showed defective heart tube formation and looping**. (A) At E9.0, wildtype embryos formed a single heart tube with normal d-loop. (B) Compared to wildtype embryos, Mesp1 (−/−) embryos had two separated heart tubes on either side of the mid-line. Sequential sections through the heart region clearly revealed normal heart tube looping in E9.0 wildtype embryos (A1–A3) and abnormal structures in E9.5 Mesp1 (−/−) embryos (B1–B3). Also neural fold closure is defective in Mesp1 (−/−) embryos (B and B1). nt, neural tube; g, gut; R, right side; L, left side. Adapted from Saga (1998) with permission

Mesp1 was firstly identified in 1996 as a novel bHLH protein appearing in the mesoderm at the early stage of mouse gastrulation (Saga et al., 1996). *In situ* hybridization and Mesp1 promoter driven beta-galactosidase gene (LacZ) studies showed that, in mouse embryonic development, the Mesp1-expressing cells were observed to ingress through the primitive streak and later in a wedge-shaped distribution in the nascent mesoderm at embryonic day 6.5–7.0 (E6.5–7.0) (Saga et al., 1999). Its initial expression was promptly down-regulated with a weaker expression observed in the presomitic mesoderm and was later observed to localize only at the base of the allantois at E7.5. Furthermore, transgenic offspring generated by crossing lacZ reporter mice with Mesp1-CRE mice indicated that, at E9.5, cells transiently expressed Mesp1 during development were mainly observed in the heart, dorsal aorta, intersomitic and cranial vessels, and the amnion contiguous to the closing foregut. Cells of all cardiac lineages including the myocardium, the endocardium, the conduction cells, and the epicardium were all β-Gal-positive (Saga et al., 1999). These results indicated Mesp1 at least marked the precursors of multipotent cardiovascular progenitors (MCPs) of both heart fields and was required for these precursor cells to depart from the primitive streak and to generate a single heart tube (Saga et al., 2000). However, Mesp1 knockout mice exhibited a morphogenetic abnormality in the heart but did not lead to absence of cardiac and vascular cells (Fig. 2), possibly due to functional compensation mediated by the massive up-regulation of its closest homologue Mesp2. In addition, the Mesp1-null embryo did not disrupt somitogenesis because of normal expression of the Mesp2 gene (Saga, 1998; Saga et al., 1999). Mesp1 and Mesp2 double-knockout embryo showed a complete defect of mesodermal migration and heart formation which confirmed the compensation effect of Mesp2 for Mesp1 (Kitajima et al., 2000).

**Figure 3:**
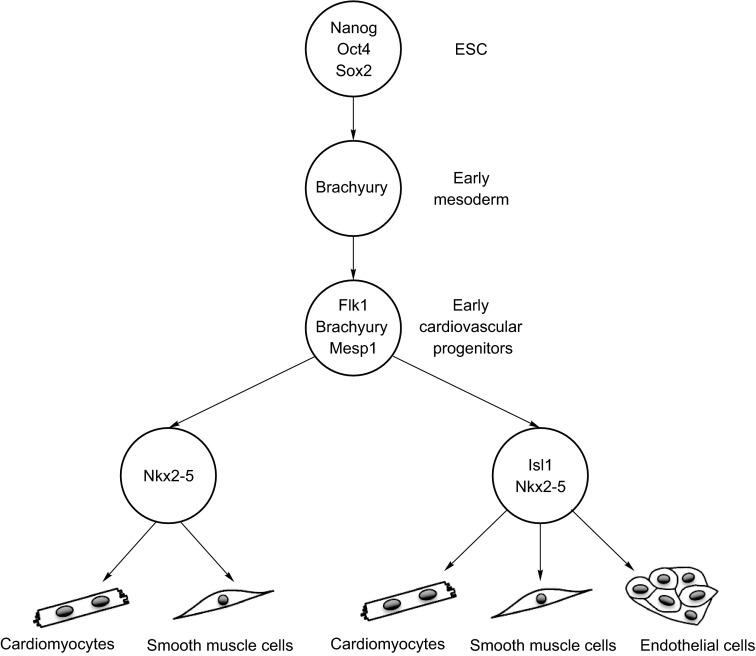
**Hierarchy model of cardiovascular specification from pluripotent stem cells**. During stem cell differentiation, Brachyury T expression marks the precardiac mesodermal cells; Mesp1-expressing cells represent early multipotent cardiovascular progenitors; Nkx2-5 and Islet1 reporter genes are used for isolating cardiovascular progenitors of the first (Nkx2-5) and the second heart fields (Nkx2-5 and Islet1). Finally, cardiovascular progenitors give rise to all three lineages of the heart: cardiomyocytes, endothelial cells, and smooth muscle cells

The transient nature of Mesp1 expression during mouse embryonic development makes it difficult to study the Mesp1-related molecular networks regulating early cardiovascular lineage specification. Luckily, differentiation of the pluripotent embryonic stem cells (ESCs) into downstream cells of the 3 embryonic germ layers, although lacking of partial *in vivo* morphogenesis, still provides an important and reliable *in vitro* model recapitulating many essential cellular and molecular events necessary in triggering lineage-specific differentiation (Kouskoff et al., 2005; Kattman et al., 2007; Murry and Keller, 2008). During embryonic development, formation of the nascent mesoderm requires the spatially and temporally regulated expression of genes involved in the Wnt, BMP, and Nodal pathways (Rossant and Tam, 2004). These factors participated in the ingress and migration of epithelial cells in the gastrulating epiblast (via epithelial-to-mesenchymal transformation (EMT)), and further regulated the expression of Brachyury T which marks the nascent mesodermal cells. Subsequent fate restriction of mesodermal precursors toward cardiovascular and hematopoietic progenitors was identified by the expression of Mesp1 and Flk1 (Wu et al., 2008). It is well-known that the heart is composed of multiple cell types (including cardiomyocytes, endothelial cells (ECs), smooth muscle cells, and cardiac fibroblasts). The cardiac cells were all traced back to two original sources of MCPs, namely first heart field (FHF) and second heart field (SHF) progenitors, with an additional contribution of neural crest cells (Fig. 3) (Bondue et al., 2011; Buckingham and Desplan, 2010). To track the earliest Mesp1-expressing cells during ESC differentiation, Bondue et al. generated Mesp1-GFP reporter ESCs and showed that these early Mesp1-expressing cells were enriched for MCPs of both heart fields, which gave rise upon differentiation to all cardiovascular cell lineages both *in vitro* and *in vivo*. Transcriptional profiling following Mesp1 overexpression demonstrated that Mesp1 rapidly activated many key genes belonging to the core cardiac transcriptional machinery (e.g., Hand2, Myocardin, Nkx2-5, Gata4, Mef2c, Tbx20, and FoxH1), and repressed genes involved in early primitive streak formation (e.g., Brachyury and FGF8) and endoderm specification (e.g., Foxa2, Gsc, Sox17, Nodal, and Cer1). Additionally, chromatin immunoprecipitation experiments showed that Mesp1 directly bound to conserved E-Box within genomic regions of several upregulated (Hand2, Myocardin, Nkx2-5, and Gata4) and downregulated genes (Foxa2, Gsc, Sox17, and Brachyury), suggesting that Mesp1 directly regulates gene transcription to induce cardiovascular specification and inhibit acquisition of other possible cell fates during this developmental stage (Bondue et al., 2008). At the same time, two other groups also reported that Mesp1 induced cardiovascular differentiation of ESCs (David et al., 2008; Lindsley et al., 2008). Mesp1 alone can induce ectopic heart tissue formation in vertebrates (David et al., 2008). Transient overexpression of Mesp1 is sufficient to direct ESCs to the cardiac mesoderm while inhibiting the development of other mesodermal lineages such as the hematopoietic lineage (Lindsley et al., 2008). A recent study using the *in vitro* ESCs differentiation model provided further evidence that Mesp1 plays a key role in specifying cardiac development. Surface markers of mesodermal progenitor cells PDGFRα, CD13, and ROR2 were elevated in the Mesp1 promoter-driven mCherry (Mesp1-mCherry) cells derived from differentiated ESCs, compared with KDR+ PDGFRα+ cardiac progenitors in which no enrichment was observed (Den Hartogh et al., 2015). NKX2.5-eGFP and Troponin T expressing cells were also up-regulated in the Mesp1-mCherry population and enhanced by inhibition of the Wnt pathway which confirmed the potent cardiovascular specific differentiation. In addition, Mesp1-mCherry derivatives also contained smooth muscle cells and endothelial cells (Den Hartogh et al., 2015). Overall, these dada indicates that Mesp1 plays a central role in regulating embryonic cardiovascular development and cardiovascular differentiation of pluripotent stem cells.

Moreover, significant effort has been made over the past two decades for direct reprogramming of fibroblasts into cardiomyocytes which are required for cardiac regenerative therapies. Recent studies showed that the efficiency for direct reprogramming of fibroblasts into cardiomyocytes could be strongly enhanced with the addition of Mesp1 (Islas et al., 2012; Fu et al., 2013; Christoforou et al., 2013). This enhancing effect for transdifferentiation of fibroblasts to cardiomyocytes further highlights the central role of Mesp1 in cardiac cell fate determination.

Some very recent studies, however, challenged the conventional view of the roles of Mesp1 in cardiovascular development. Chan et al. showed that, in addition to promoting cardiac differentiation, Mesp1 promoted hematopoietic differentiation and skeletal myogenic differentiation of ESCs in different induction conditions. Lineage tracing studies in mice also showed that the majority of yolk sac and certain adult hematopoietic cells were actually derived from Mesp1+ cells. It is thus proposed that Mesp1 specifies different lineage derivatives in a context-dependent manner (Chan et al., 2013). This study challenged the previous view of Mesp1 being a master regulator mostly for specifying cardiovascular development. Another very recent study also further clarified a question of whether Mesp1-positive progenitors represent the primitive MCPs common to both FHF and SHF. Using temporal clonal analysis of Mesp1-expressing cells, Lescroart et al. provided compelling evidence that Mesp1 marked distinct classes of cardiovascular progenitors (FHF Mesp1+ progenitors and SHF Mesp1+ progenitors) differentiating into restricted lineages at different time points during gastrulation. FHF Mesp1+ progenitors were unipotent and different from the SHF Mesp1+ progenitors which were either unipotent or bipotent (Lescroart et al., 2014). All these data suggested that detailed molecular processes and the roles of Mesp1 in early cardiovascular lineage specification still require further investigation.

Overall, current research data indicates that the mesoderm transcription factor Mesp1 seems to situate in the core of the complicated regulatory network by generating cardiovascular progenitors and prompting gastrulation development. Mesp1 is transiently expressed in the anterior mesoderm from the onset of gastrulation prior to cardiac crescent formation. MESP1-expressing progenitors generated almost all cells of the heart including myocardium, endocardium, epicardium, and cells of the conduction system. Mesp1 directly and/or indirectly regulated the expression of the majority of key cardiovascular transcription factors including Hand2, Myocardin, Nkx2-5, Gata4, Mef2c, Foxc1, and Foxc2, thus acting as a master regulator and the earliest marker of cardiovascular development in vertebrates and cardiovascular differentiation in mammalian pluripotent stem cells.

## FUNCTIONAL ROLE OF MESP2 IN EMBRYONIC MESODERMAL DEVELOPMENT

In 1997, Saga et al. isolated a novel gene encoding bHLH protein Mesp2, which has an almost identical bHLH motif to that of Mesp1 (93% amino acid identity) and contains a unique region at the carboxyl-terminus (Saga et al., 1997). At E6.5–7.0, Mesp2 was weakly expressed in the early mesoderm, in a pattern very similar to that of Mesp1 (Kitajima et al., 2000). Since E8.0, Mesp2 was dynamically expressed in presomitic mesoderm of mouse embryos, which overlapped completely with the Mesp1 expression domain (Saga et al., 1997).

**Figure 4:**
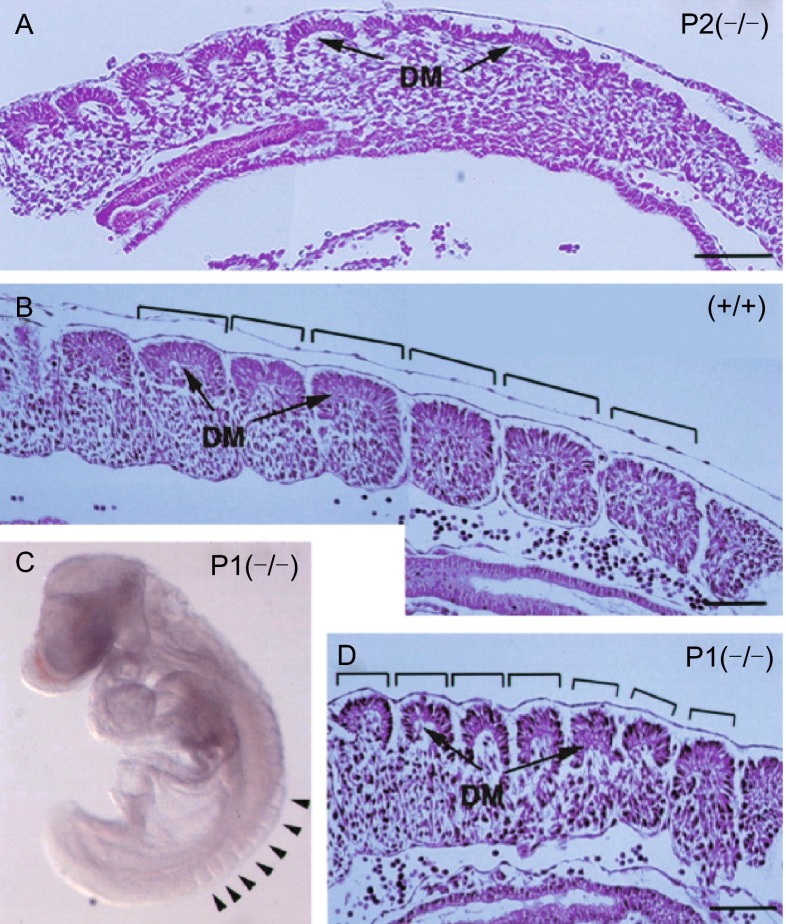
**Mesp2-null embryos exhibited defective segmentation in somitogenesis**. (A) In Mesp2 (−/−) embryos at E9.5, segmentation of somites is defective, while differentiation into dermomyotome (DM) proceeds. Compared to wildtype (+/+) embryos at the same developmental stage (B), Mesp1 (−/−) embryos showed differentiation into dermomyotome and sclerotome with segmentation exhibiting reduced segmental width (indicated by the width of brackets) (C and D). DM, dermomyotome. Bars, 100 μm. Adapted from Saga (1998) with permission

Disruption of the Mesp2 gene in mice impaired segmentation of the somitic mesoderm and resulted in loss of the rostral properties within the somite (Fig. 4). Consequently, the Mesp2 knockout mutant pups exhibited fused vertebral columns and caudal truncation, and died within 20 min after birth (Saga et al., 1997). Mesp2 gene mutations are also found in human patients with spondylocostal dysostosis which is a rare, heritable axial skeleton growth disorder (Whittock et al., 2004).

Somite formation starts from E8.0 and ends at E13.0 in mice. During somitogenesis, the presomitic mesoderm is sequentially subdivided into blocks of cells to form somites. Prospective segmentation site is defined by Notch oscillators, while pace of segmentation is regulated by Fgf oscillators (Saga, 2012). Actually the most reliable molecular marker of segmentation initiation is the transcriptional activation of Mesp2. Tbx6 and Notch signaling are required for the induction of Mesp2 expression (Yasuhiko et al., 2006), while up-regulation of Mesp2 protein in turn leads to the suppression of Tbx6 expression post-translationally via rapid protein degradation through the ubiquitin-proteasome pathway. Therefore the expression of Mesp2 is tightly regulated by a negative feedback loop *in vivo* (Oginuma et al., 2008).

Activation of Notch signaling during somitogenesis can be visualized by immunohistochemistry using a specific antibody to the processed Notch intracellular domain (NICD). At the initial phase of Mesp2 expression in somitogenesis, Mesp2 exhibited a partially co-expressing pattern with NICD as reflected by double immunohistochemistry (Morimoto et al., 2005). Mesp2 suppressed itself through a feedback loop and suppressed Notch activity by destabilizing Mastermind-like 1 (MamL1), one of the core components of the nuclear NICD complex, at the post-transcriptional level via pathways other than the proteasome pathway (Sasaki et al., 2011). As expression levels of Mesp2 increase, the overlapping domain of NICD and Mesp2 gradually reduced. Ultimately, the caudal half of Mesp2 expression gradually shrinked, leaving the rostral half band intact. The Mesp2 and NICD expression domains completely separated from each other and formed a clear boundary, which produced the next segmental boundary (Morimoto et al., 2005). Mesp2 also activated its target genes EphA4, which led to the generation of the morphological border (Nakajima et al., 2006; Saga, 2007).

Notch signaling activity is also a required determinant of the caudal identity of the somite. Mesp2 suppressed Notch activity in the rostral compartment by suppressing Notch ligand Dll1 and repressed the caudal gene Uncx4.1 to define rostral identity (Takahashi et al., 2000). Mesp2 also induced the expression of Ripply 1 and 2, which in turn played roles in restricting the expression domain of Mesp2 by suppressing the activity of Tbx6. This negative feedback loop is essential for the generation of the rostro-caudal polarity periodically (Takahashi et al., 2010).

In summary, Mesp2 is required for the normal segmentation of somites and generation of the rostro-caudal polarity of somites.

## FUNCTIONAL REDUNDANCY OF MESP1 AND MESP2 IN EMBRYONIC MESODERMAL DEVELOPMENT

Segmented somites were detected in Mesp1 deficient embryos, indicating that Mesp1 was not essential for somitogenesis (Takahashi et al., 2005). However, during somitogenesis, the Mesp1 gene was also expressed at the same time and sites as those of Mesp2. Thus, it is speculated that Mesp1 might play a role in somitogenesis. Furthermore, in Mesp2 null embryos, there was differentiation into dermomyotome and sclerotome and delayed irregular segmentation of the dermomyotome without obvious segmentation in the sclerotome (Fig. 4) (Saga et al., 1997). Chimera analysis showed Mesp1/Mesp2 double-knockout cells were not able to undergo epithelialization, whereas Mesp2 single-knockout cells were integrated into epithelial somites and dermomyotome occasionally (Takahashi et al., 2005). Taken together, Mesp1 does contribute to the epithelialization of dermomyotome observed in Mesp2-null embryos (Saga et al., 1997).

**Figure 5:**
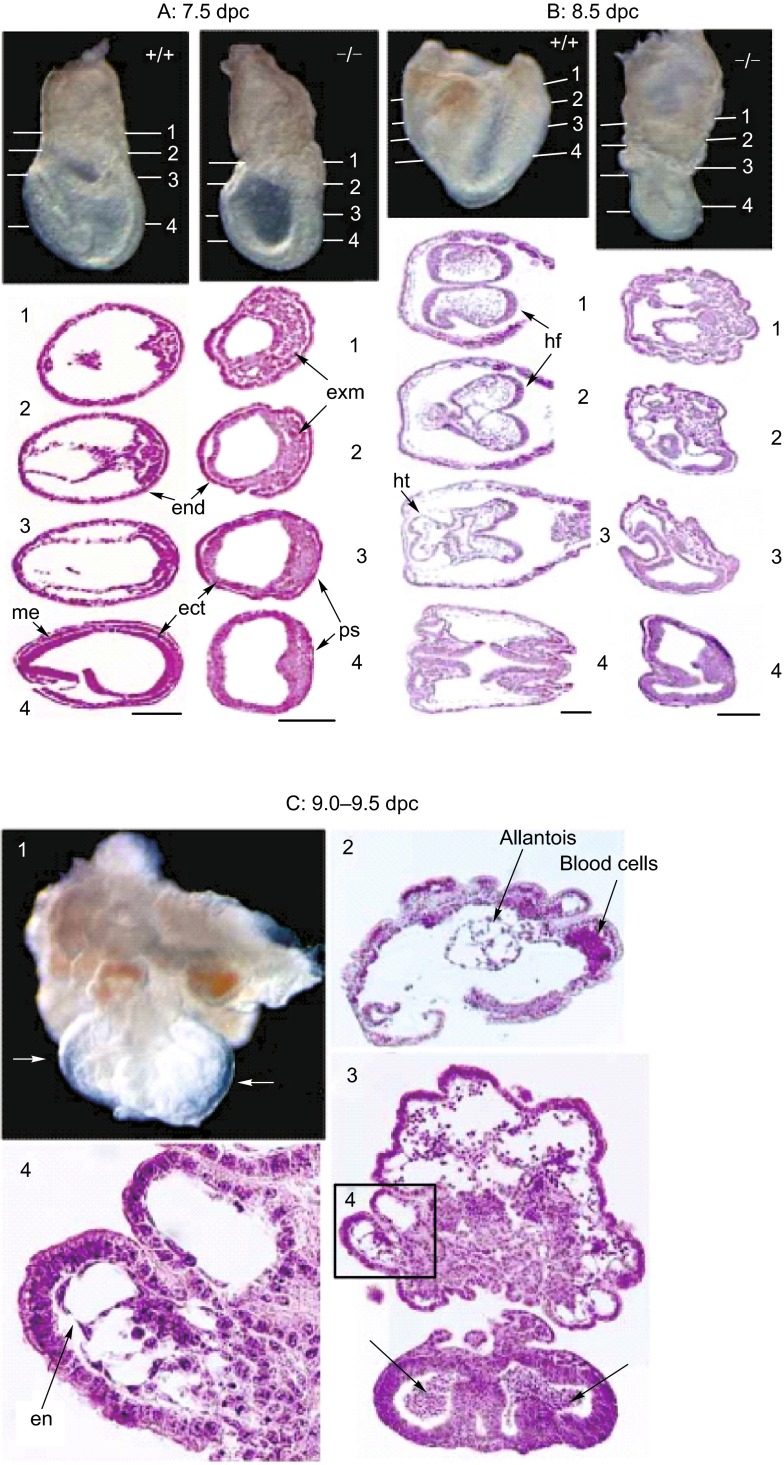
**The Mesp1/Mesp2 double-knockout embryos exhibited defective development of the embryonic mesoderm**. (A) At E7.5, in Mesp1/Mesp2 double-knockout embryo, no clear mesodermal cell layer is observed between endodermal and ectodermal layers; dense cell accumulated in primitive streak and many mesodermal cells accumulated in extraembryonic region. (B) Similarly, at E8.5, although heart tube and mesenchymal cells in the headfold are developed in the wildtype embryo, mesodermal cells are still not migrated in the Mesp1/Mesp2 double-knockout embryos. (C) E9.0–9.5 mutant embryos show extensive pile-up of cell layers in the extraembryonic region and smaller embryonic region with no trunk structure such as the heart, somites or gut developed. exm, extraembryonic mesoderm; end, endoderm; ect, ectoderm; me, embryonic mesoderm; ps, primitive streak; hf, headfold; ht, heart; en, endothelial cells. Scale bars, 100 μm. Adapted from Kitajima et al. (2000) with permission

Overexpression of Mesp1 is sufficient to generate multipotent cardiovascular progenitor from ESCs *in vitro* (Bondue et al., 2008; David et al., 2008; Lindsley et al., 2008). Meanwhile, in Mesp2 null mice, no notable developmental defect was observed prior to somitogenesis, which was consistent with the low expression level of Mesp2 during the early gastrulation stage. However, in Mesp1 deficiency mice, mesodermal precursors finally migrated into the heart field, differentiated and contributed to defective heart morphogenesis, while in Mesp1/Mesp2 double-knockout embryos there was an accumulation of nonmigrating cells in the primitive streak and complete failure to form cardiac mesoderm (Fig. 5) (Saga et al., 1999; Kitajima et al., 2000). Mesp2 was weakly expressed at E6.5–7.0 in a pattern very similar to Mesp1 expression, but expressed longer in Mesp1 (−/−) embryos compared to wildtype. The initial deficiency in Mesp1 null embryos may be partially rescued by the later induced and prolonged expression of Mesp2. Studies by Lindsley et al. further showed that transient expression of Mesp2 induced expression of EMT genes in DKK treated cultures (Lindsley et al., 2008). Taken together, Mesp2 can induce mesoderm and EMT in ESCs, and compensate for differentiation and migratory defects in Mesp1-deficient embryos.

In conclusion, Mesp1 and Mesp2 not only have their respective specific functions during mammalian cardiac development and somitogenesis, but also exhibit functional redundancy in these developmental events.

## FUTURE RESEARCH

In addition to genetic approaches, integration of cellular, molecular, and biochemical methods using both animal and ESC models will further help elucidate the precise regulatory mechanisms underlying human heart development and somitogenesis. Establishing a reliable *in vitro* developmental model using human ESCs will be critical for further elucidating the specific and overlapping roles of Mesp1 and Mesp2 in early mesodermal development of humans.

Further, it will be important to identify interacting partner proteins of Mesp1 and Mesp2, which could be able to form a protein complex together with Mesp1 or Mesp2, functioning in strict post-translational control or modulating the transcriptional activities by determining specific target sequences. A better delineation of the molecular events involving Mesp1 and Mesp2 will not only contribute to an improved understanding of mesodermal development, but will also be helpful to increase the production of specific cells (e.g. cardiomyocytes and myoblasts) from the pluripotent stem cells for future cellular therapy and drug screening.

## References

[CR1] Bondue A, Lapouge G, Paulissen C, Semeraro C, Iacovino M, Kyba M, Blanpain C (2008). Mesp1 acts as a master regulator of multipotent cardiovascular progenitor specification. Cell Stem Cell.

[CR2] Bondue A, Tännler S, Chiapparo G, Chabab S, Ramialison M, Paulissen C, Beck B, Harvey R, Blanpain C (2011). Defining the earliest step of cardiovascular progenitor specification during embryonic stem cell differentiation. J Cell Biol.

[CR3] Buckingham M, Desplan C (2010). Developmental mechanisms, patterning and evolution. Curr Opin Genet Dev.

[CR4] Chan SS, Shi X, Toyama A, Arpke RW, Dandapat A, Iacovino M, Kang J, Le G, Hagen HR, Garry DJ (2013). Mesp1 patterns mesoderm into cardiac, hematopoietic, or skeletal myogenic progenitors in a context-dependent manner. Cell Stem Cell.

[CR5] Christoforou N, Chellappan M, Adler AF, Kirkton RD, Wu T, Addis RC, Bursac N, Leong KW (2013). Transcription factors MYOCD, SRF, Mesp1 and SMARCD3 enhance the cardio-inducing effect of GATA4, TBX5, and MEF2C during direct cellular reprogramming. PLOS ONE.

[CR6] David R, Brenner C, Stieber J, Schwarz F, Brunner S, Vollmer M, Mentele E, Müller-Höcker J, Kitajima S, Lickert H (2008). MesP1 drives vertebrate cardiovascular differentiation through Dkk-1-mediated blockade of Wnt-signalling. Nat Cell Biol.

[CR7] Den Hartogh SC, Schreurs C, Monshouwer Kloots JJ, Davis RP, Elliott DA, Mummery CL, Passier R (2015). Dual reporter MESP1 mCherry/w-NKX2-5 eGFP/w hESCs enable studying early human cardiac differentiation. Stem Cells.

[CR8] Fu J, Stone NR, Liu L, Spencer CI, Qian L, Hayashi Y, Delgado-Olguin P, Ding S, Bruneau BG, Srivastava D (2013). Direct reprogramming of human fibroblasts toward a cardiomyocyte-like state. Stem Cell Rep.

[CR9] Islas JF, Liu Y, Weng K, Robertson MJ, Zhang S, Prejusa A, Harger J, Tikhomirova D, Chopra M, Iyer D (2012). Transcription factors ETS2 and MESP1 transdifferentiate human dermal fibroblasts into cardiac progenitors. Proc Natl Acad Sci USA.

[CR10] Kattman SJ, Adler ED, Keller GM (2007). Specification of multipotential cardiovascular progenitor cells during embryonic stem cell differentiation and embryonic development. Trends Cardiovasc Med.

[CR11] Kitajima S, Takagi A, Inoue T, Saga Y (2000). MesP1 and MesP2 are essential for the development of cardiac mesoderm. Development.

[CR12] Kouskoff V, Lacaud G, Schwantz S, Fehling HJ, Keller G (2005). Sequential development of hematopoietic and cardiac mesoderm during embryonic stem cell differentiation. P Natl Acad Sci USA.

[CR13] Lescroart F, Chabab S, Lin X, Rulands S, Paulissen C, Rodolosse A, Auer H, Achouri Y, Dubois C, Bondue A et al (2014) Early lineage restriction in temporally distinct populations of Mesp1 progenitors during mammalian heart development. Nat Cell Biol 16:829–840 10.1038/ncb3024 PMC6984965 25150979

[CR14] Lindsley RC, Gill JG, Murphy TL, Langer EM, Cai M, Mashayekhi M, Wang W, Niwa N, Nerbonne JM, Kyba M (2008). Mesp1 coordinately regulates cardiovascular fate restriction and epithelial-mesenchymal transition in differentiating ESCs. Cell stem Cell.

[CR15] Morimoto M, Takahashi Y, Endo M, Saga Y (2005). The Mesp2 transcription factor establishes segmental borders by suppressing Notch activity. Nature.

[CR16] Murry CE, Keller G (2008). Differentiation of embryonic stem cells to clinically relevant populations: lessons from embryonic development. Cell.

[CR17] Nakajima Y, Morimoto M, Takahashi Y, Koseki H, Saga Y (2006). Identification of Epha4 enhancer required for segmental expression and the regulation by Mesp2. Development.

[CR18] Oginuma M, Niwa Y, Chapman DL, Saga Y (2008). Mesp2 and Tbx6 cooperatively create periodic patterns coupled with the clock machinery during mouse somitogenesis. Development.

[CR19] Rossant J, Tam PP (2004). Emerging asymmetry and embryonic patterning in early mouse development. Dev Cell.

[CR20] Saga Y (1998). Genetic rescue of segmentation defect in MesP2-deficient mice by MesP1 gene replacement. Mech Dev.

[CR21] Saga Y (2007). Segmental border is defined by the key transcription factor Mesp2, by means of the suppression of Notch activity. Dev Dyn.

[CR22] Saga Y (2012). The mechanism of somite formation in mice. Curr Opin Genet Dev.

[CR23] Saga Y, Hata N, Kobayashi S, Magnuson T, Seldin MF, Taketo MM (1996). MesP1: a novel basic helix-loop-helix protein expressed in the nascent mesodermal cells during mouse gastrulation. Development.

[CR24] Saga Y, Hata N, Koseki H, Taketo MM (1997). Mesp2: a novel mouse gene expressed in the presegmented mesoderm and essential for segmentation initiation. Gene Dev.

[CR25] Saga Y, Miyagawa-Tomita S, Takagi A, Kitajima S, Miyazaki JI, Inoue T (1999). MesP1 is expressed in the heart precursor cells and required for the formation of a single heart tube. Development.

[CR26] Saga Y, Kitajima S, Miyagawa-Tomita S (2000). Mesp1 expression is the earliest sign of cardiovascular development. Trends Cardiovasc Med.

[CR27] Sasaki N, Kiso M, Kitagawa M, Saga Y (2011). The repression of Notch signaling occurs via the destabilization of mastermind-like 1 by Mesp2 and is essential for somitogenesis. Development.

[CR28] Takahashi Y, Koizumi K, Takagi A, Kitajima S, Inoue T, Koseki H, Saga Y (2000). Mesp2 initiates somite segmentation through the Notch signalling pathway. Nat Genet.

[CR29] Takahashi Y, Kitajima S, Inoue T, Kanno J, Saga Y (2005). Differential contributions of Mesp1 and Mesp2 to the epithelialization and rostro-caudal patterning of somites. Development.

[CR30] Takahashi J, Ohbayashi A, Oginuma M, Saito D, Mochizuki A, Saga Y, Takada S (2010). Analysis of Ripply1/2-deficient mouse embryos reveals a mechanism underlying the rostro-caudal patterning within a somite. Dev Biol.

[CR31] Whittock NV, Sparrow DB, Wouters MA, Sillence D, Ellard S, Dunwoodie SL, Turnpenny PD (2004). Mutated MESP2 causes spondylocostal dysostosis in humans. Am J Hum Genet.

[CR32] Wu SM, Chien KR, Mummery C (2008). Origins and fates of cardiovascular progenitor cells. Cell.

[CR33] Yasuhiko Y, Haraguchi S, Kitajima S, Takahashi Y, Kanno J, Saga Y (2006). Tbx6-mediated Notch signaling controls somite-specific Mesp2 expression. Proc Natl Acad Sci USA.

